# Evaluation of tenascin-C by tenatumomab in T-cell non-Hodgkin lymphomas identifies a new target for radioimmunotherapy

**DOI:** 10.18632/oncotarget.23919

**Published:** 2018-01-03

**Authors:** Giuseppe Gritti, Andrea Gianatti, Fiorella Petronzelli, Rita De Santis, Chiara Pavoni, Riccardo Lorenzo Rossi, Laura Cattaneo, Luigi Giusto Spagnoli, Silvia Ferrari, Andrea Rossi, Anna Maria Barbui, Alessandro Rambaldi

**Affiliations:** ^1^ Hematology Unit, Ospedale Papa Giovanni XXIII, Bergamo, Italy; ^2^ Pathology Unit, Ospedale Papa Giovanni XXII, Bergamo, Italy; ^3^ Sigma Tau S.p.A. Biotech Products R and D, Pomezia, Italy; ^4^ Bioinformatics, Istituto Nazionale Genetica Molecolare, Milan, Italy; ^5^ Department of Biomedicine and Prevention, Università di Roma Tor Vergata, Rome, Italy; ^6^ Department of Oncology and Oncohematology, Università degli Studi di Milano, Milan, Italy

**Keywords:** T-cell non-Hodgkin lymphoma, peripheral T-cell lymphoma, cutaneous T-cell lymphoma, tenascin-C, radioimmunotherapy

## Abstract

The clinical outcome of T-cell non-Hodgkin lymphoma (NHL) is poor and innovative treatments are needed. Tenascin-C is a large extracellular glycoprotein not expressed under physiological conditions, but overexpressed in cancer. Aim of the study was to evaluate tenascin-C expression within pathologic tissue of T-cell NHL and determine its clinical significance. We used an immunohistochemistry approach using the anti-tenascin-C monoclonal antibody Tenatumomab in 75 systemic T-cell NHL (including 72 mature and 3 precursor T-cell NHL), and 25 primary cutaneous T-cell NHL. Data were analyzed in terms of staining intensity, proportion of involved areas and histologic pattern, and results were correlated with clinical characteristics and outcome. Ninety-three percent of the cases were tenascin-C positive and 59% of systemic diseases were characterized by a predominant involvement (>50%). Stromal expression was detected in all the cases while vascular and vascular plus cytoplasmic expression was present in 49% and 23%. The constant overexpression of the tenascin-C gene was observed in two independent publicly available T-cell NHL gene expression datasets. In conclusions, tenascin-C represents an attractive target that sets the rationale to investigate the therapeutic activity of radiolabeled Tenatumomab in T-cell NHL.

## INTRODUCTION

Over the past few years several new therapeutic options have become available for relapsed or refractory B-cell non-Hodgkin lymphomas (NHL) and Hodgkin lymphoma [1, 2], but only marginal progress if any has been achieved for mature T-cell NHL [3–6]. T-cell NHL is a heterogeneous and relatively rare group of malignancies representing 7–15% of all NHL [7]. It includes systemic NHL, deriving from both precursor (lymphoblastic lymphoma) and mature T-cell (peripheral T-cell lymphoma, PTCL), and primary cutaneous mature T-cell lymphoma (CTCL). While the latter category shows a variable outcome with patients with aggressive histologies and/or advanced stage suffering of adverse outcome, prognosis of systemic T-cell NHL is generally poor with conventional treatment, with the notable exception of low-risk, ALK positive, anaplastic large cell lymphoma (ALCL) patients [8, 9]. Thus, identification of new therapeutic strategies in T-cell lymphoma is a major unmet need.

Tenascin-C is a large hexameric glycoproteins found in embryonic and adult extracellular matrices [10]. It can undergo alternative splicing resulting in large (up to 320 kDa monomer) or small (220 kDa monomer) isoforms. The large tenascin-C variant is preferentially expressed in malignant tissues, is spatially and temporally related to tumor neovascularization and may exert anti-adhesive [11–13], and immunosuppressive activities [14]. The presence of tenascin-C within tumor infiltrated tissues made it as an attractive candidate for antibody mediated therapy. Based on the remarkable expression of tenascin-C within lymph nodes of patients with B-cell NHL and Hodgkin lymphoma, radio-immunotherapy (RIT) approaches have been developed [15–19], and phase I clinical trials in different settings have shown both feasibility and therapeutic efficacy of tenascin-targeting therapeutic approaches [20, 21]. Tenatumomab (ST2146, Sigma Tau S.p.A. R&D, Pomezia, Italy) is a murine IgG2b/k antibody recognizing an epitope within the EGF-like repeats of human tenascin, shared by both small and large tenascin isoforms [22–24]. Tenatumomab conjugated with ^131^I has been developed for RIT applications, and it is currently in phase I clinical development in tenascin-C expressing cancer (NCT02602067). In the present study, we evaluate the presence of tenascin-C using Tenatumomab antibody in a series of diagnostic biopsies of T-cell NHL patients.

## RESULTS

### Immunohistochemical expression of tenascin-C

**Figure 1:**
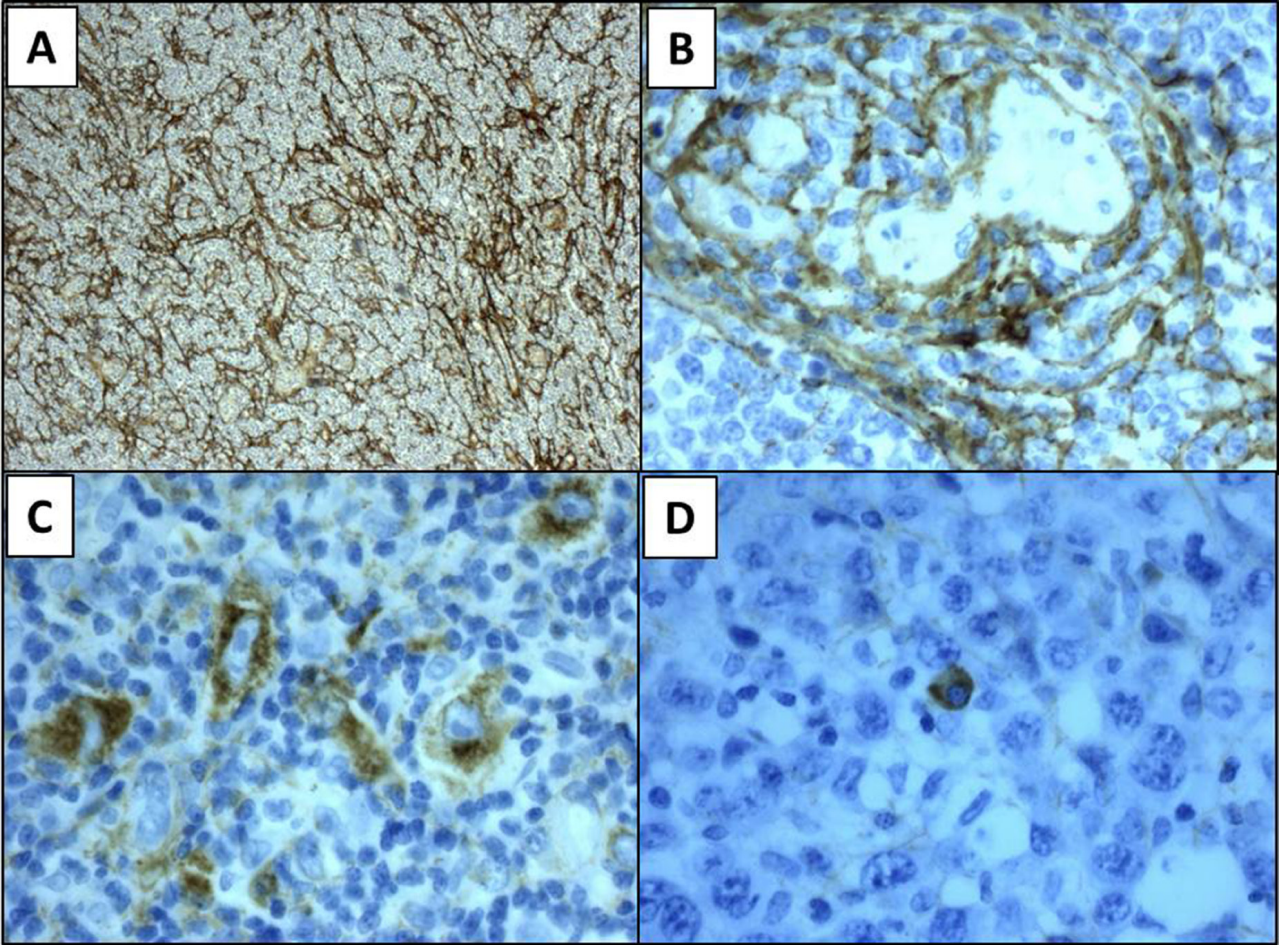
Representative panels of pattern tenascin-C expression Stromal pattern of expression (panel **A**), vascular (**B**), cytoplasmic in neoplastic lymphocytes (**C**) and in normal plasma cell (**D**). Magnification 10× (A), 40× (B–D).

**Figure 2:**
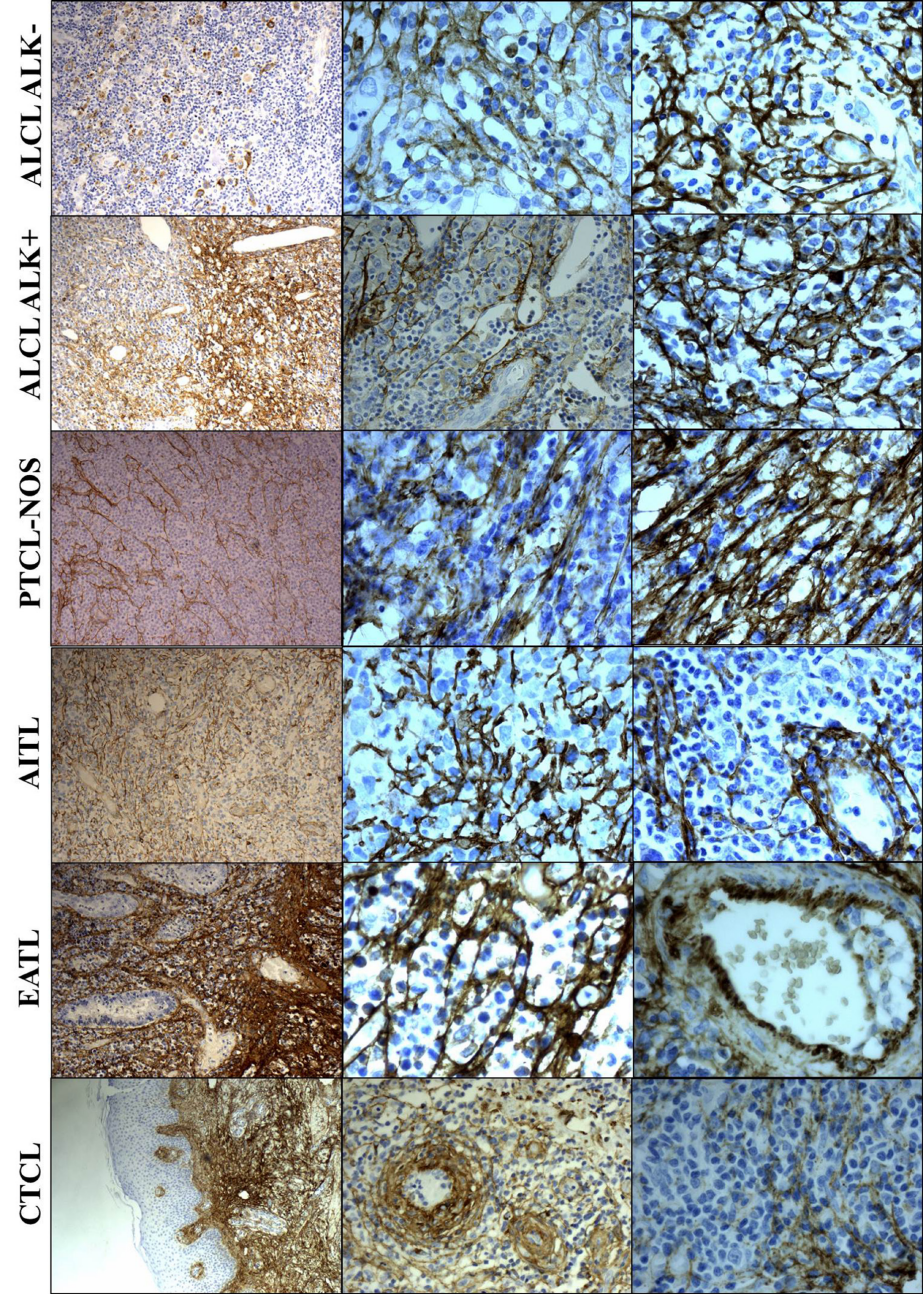
Tenascin-C expression in the different T-cell NHL subtypes Legend: ALCL: anaplastic large cell lymphoma; ALK: anaplastic large cell lymphoma kinase; PTCL: peripheral T-cell lymphoma; NOS: not otherwise specified; AITL: angioimmunoblastic lymphoma; EATL: enteropathy-associated T-cell lymphoma; CTCL: cutaneous T-cell lymphoma. Magnification: 10x and 40x.

total of 100 diagnostic biopsies from patients with T-cell NHL were evaluated for immunohistochemical (IHC) tenascin-C expression with Tenatumomab. Of the 100 patients evaluated, 75 cases had a diagnosis of systemic T-cell NHL, including 72 PTCL and 3 of precursor T-cell NHL, while 25 were CTCL. Among systemic T-cell NHL, 40 patients had a diagnosis of ALCL (ALK negative, *N =* 21 or ALK positive, *N =* 19), PTCL-NOS (*N =* 20), AITL (*N =* 9), T-cell lymphoblastic lymphoma (*N =* 3), enteropathy-associated T-cell lymphoma (*N =* 2), hepatosplenic T-cell lymphoma (*N =* 1). Regarding CTCL, the most common histology was mycosis fungoides/Sézary syndrome (MF/SS, *N =* 14) followed by primary cutaneous anaplastic large cell lymphoma (pcALCL, *N =* 6) and CD4+ small/medium cell primary cutaneous lymphoma (pcSMPTCL, *N =* 5). Data summary of the expression of tenascin-C in T-cell NHL is presented in Table 1. Tenatumomab revealed the presence of tenascin-C in nearly all cases evaluated (93%). A high proportion of tenascin-C positive areas in pathologic samples (> 50%) were shown in half of the patients (Table 1). There was a significantly different distribution among the specific histology (*P* = 0.0043). Most of the systemic T-cell NHL (59%) were characterized by a diffuse expression (> 50%) of tenascin-C, while this was the case only in a minority of patients with MF/SS (21%) and pcALCL (50%). In systemic T-cell NHL, diffuse expression (> 50%) was detected in 76% of the ALCL ALK negative patients, 78% of AITL and 53% ALCL ALK positive. A lower (< 50%) tenascin-C involvement was more frequently detected in PTCL NOS (65%), MF/SS (79%) and pcALCL (50%). The staining intensity was not significantly different among histologies (*P* = 0.5791), but a higher staining intensity (grade 2–3) was more common in AITL (67%), ALCL ALK positive (68%) and negative (57%), MF/SS (57%) and pcALCL (67%). A direct correlation was found between Tenatumomab staining intensity and proportion of involved areas by tenascin-C (*P* < 0.001). The histological pattern of tenascin-C expression was recorded (Figure 1 and Table 1). As tenascin-C is a structural component of the extracellular matrix, a stromal histological pattern of expression was observed in all the positive cases. In 50% of the samples, tenascin-C was present strictly adjacent to vascular structures, but not into endothelial cells. In a minority of the cases (23%), the protein was also intracellular (cytoplasmic). Vascular pattern was revealed in 56% of systemic T-cell NHL and 20% of CTCL (*P* = 0.0026). High prevalence of vascular expression was shown in AITL (100%), ALCL ALK negative (74%), PTCL NOS (44%) and ALCL ALK POSITIVE (42%). Cytoplasmic positivity for tenascin-C was present only in cases showing a vascular expression and was recorded nearly exclusively in PTCL (20 of 21 positive cases), in particular in AITL (44%), ALCL ALK negative (37%) and positive (32%). A representative panel of immune staining showing the different pattern of expression of tenascin-C for the main T-cell NHL subtype is shown in Figure 2.

**Table 1:** Summary of staining characteristics

Histology	*N*	Staining intensity				Proportion of involved areas (%)				Pattern of expression			
		0	1	2	3	0- 25	26-50	51-75	76-100	Negative	Stromal only	Vascular+ Stromal only	Vascular+ Stromal+ Cytoplasm
**Precursor (lymphoblastic) T-cell NHL**	3	0	1	1	1	1	0	2	0	0	2	1	0
*Peripheral T-cell lymphoma, not otherwise specified*	*20*	*2*	*10*	*5*	*3*	*5*	*9*	*5*	*1*	*2*	*10*	*5*	*3*
*Anaplastic large cell lymphoma, ALK negative*	*21*	*2*	*7*	*7*	*5*	*2*	*2*	*14*	*3*	*2*	*5*	*7*	*7*
*Anaplastic large cell lymphoma, ALK positive*	*19*	*0*	*6*	*7*	*6*	*0*	*8*	*9*	*2*	*0*	*11*	*2*	*6*
*Angioimmunoblastic T-cell lymphoma*	*9*	*0*	*3*	*5*	*1*	*0*	*2*	*6*	*1*	*0*	*0*	*5*	*4*
*Enteropathy-associated T-cell lymphoma*	*2*	*1*	*0*	*0*	*1*	*1*	*0*	*0*	*1*	*1*	*0*	*1*	*0*
*Hepatosplenic T-cell lymphoma*	*1*	*0*	*0*	*1*	*0*	*0*	*1*	*0*	*0*	*0*	*1*	*0*	*0*
**PTCL (subtotal)**	72	5	26	25	16	8	22	34	8	5	27	20	20
*Mycosis fungoides*	*13*	*1*	*4*	*3*	*5*	*1*	*9*	*3*	*0*	*1*	*11*	*0*	*1*
*Primary cutaneous anaplastic large cell lymphom*a	*6*	*0*	*2*	*4*	*0*	*1*	*2*	*3*	*0*	*0*	*2*	*4*	*0*
*CD4*^*+*^ *small/medium cell primary cutaneous lymphoma*	*5*	*1*	*0*	*3*	*1*	*3*	*2*	*0*	*0*	*1*	*4*	*0*	*0*
*Sézary syndrome*	*1*	*0*	*1*	*0*	*0*	*0*	*1*	*0*	*0*	*0*	*1*	*0*	*0*
**CTCL (subtotal)**	25	2	7	10	6	5	14	6	0	2	18	4	1
**Total**	100	7	34	36	23	14	36	42	8	7	47	25	21

Legend: NHL: non-Hodgkin lymphoma; PTCL: peripheral T-cell lymphoma; CTCL: cutaneous T-cell lymphoma.

### Validation of immunohistochemical expression of tenascin-C by digital image analysis

Since manual determination of tenascin-C involvement may be subject to inter-investigator variability, we adopted a digital image analysis system to evaluate the actual proportion of positive areas in pathologic samples. To compare data with the manual determination, the calculated proportion of involved areas was categorized according to the same scoring system. Summary of the results are presented in Supplementary Table 1. A high concordance (84%, Weighted Kappa Coefficient = 0.83) between digital and manual scoring was present, with 7 cases underscored and 9 up scored by automated image analysis. These differences, however, did not resulted in a different distribution of tenascin-C positivity among disease group (systemic vs cutaneous) or among specific histologies.

### Correlation with clinical data

Full baseline clinical characteristics and follow-up were available for the 75 systemic T-cell NHL patients, while only data about diagnosis and survival were available for CTCL. The median age of systemic T-cell NHL was 54 years (range 14–85) with 39% of the patients older than 60 years and a male prevalence (57%). Clinical stage was advanced (Ann Arbor III–IV) in the majority of the cases (68%). The international prognostic score (IPI) was low, intermediate-low, intermediate-high and high in 40%, 15%, 22% and 23% respectively. The 5-year OS and PFS were 75% and 59% respectively for ALCL ALK positive, while for the remaining cases of systemic T-cell NHL were 30% and 26%. In CTCL, the 5-year OS was 51% for MF/SS, 100% for pcALCL and 100% for pcSMPTCL (Supplementary Figure 1). No correlation was found between both staining intensity and pattern of expression with the baseline clinical characteristics, including age (*P* = 0.8479 and *P* = 0.3498), stage (*P* = 0.6146 and *P* = 0.3227), LDH (*P* = 0.1179 and *P* = 0.1560), IPI (*P* = 0.2637 and *P* = 0.0871). Conversely, an association was found between proportion of involved areas and LDH (*P* = 0.0088) and IPI (*P* = 0.0328). Among the 17 high IPI patients, 3 (18%) did not express tenascin-C, 8 (47%) expressed staining intensity of 1, 6 (35%) a lower proportion of involved areas (< 50%) and 12 (71%) a vascular pattern of expression. Most of these high-risk patients were PTCL NOS (*N =* 6, 35%) and ALCL ALK negative (*N =* 5, 29%). Multivariate models adjusted for age, gender and IPI were calculated to analyze the impact of the histologic characteristic of tenascin-C expression with PFS and OS in the 75 systemic T-cell NHL patients (Table 2). Staining intensity nor proportion of involved areas resulted significantly associated to prognosis, while patients with a vascular pattern of tenascin-C positivity were characterized by a better OS (*P* = 0.0228). All these results were confirmed as well by using the proportion of involved areas calculated by digital image analysis (Supplementary Table 2).

**Table 2:** Multivariate analysis for PFS and OS in systemic T-cell NHL patients

Factors	Multivariate for PFS				Multivariate for OS			
	HR (95% Cl)	*P* value	HR (95% Cl)	*P* value	HR (95% Cl)	*P* value	HR (95% Cl)	*P* value
**Proportion of involved areas (%)**								
0-50	1.00				1.00			
51-100	0.53 (0.27–1.03)	0.0627			0.55 (0.27–1.12)	0.1011		
**Pattern of expression**								
Negative or Stromal only			1.00				1.00	
Vascular plus Stromal			0.52 (0.25–1.08)	0.0785			0.41 (0.19–0.88)	0.0228
**Age**								
≤ 60	1.00		1.00		1.00		1.00	
> 60	1.56 (0.70–3.51)	0.2800	1.31 (0.58–2.94)	0.5158	1.92 (0.83–4.42)	0.1256	1.59 (0.71–3.54)	0.2591
**Gender**								
Female	1.00		1.00		1.00		1.00	
Male	1.17 (0.59–2.31)	0.6506	1.33 (0.66–2.66)	0.4218	1.54 (0.75–3.14)	0.2366	1.81 (0.87–3.80)	0.1144
**IPI**								
Low	1.00		1.00		1.00		1.00	
Interm-Low	1.71 (0.61–4.81)	0.3121	1.88 (0.68–5.22)	0.226	1.96 (0.66–5.79)	0.2239	2.23 (0.77–6.47)	0.1387
Interm-High	1.81 (0.67–4.92)	0.2446	2.39 (0.91–6.29)	0.0774	1.82 (0.61–5.42)	0.2855	2.36 (0.83–6.75)	0.1089
High	**4.10 (1.57–10.68)**	**0.0039**	**5.99 (2.12–16.92)**	**0.0007**	**5.10 (1.92–13.54)**	**0.0011**	**8.41 (2.93–24.14)**	**<.0001**

Legend: NHL: non-Hodgkin lymphoma; PFS: progression free survival; OS: overall survival.

### Gene expression of tenascin-C

**Figure 3:**
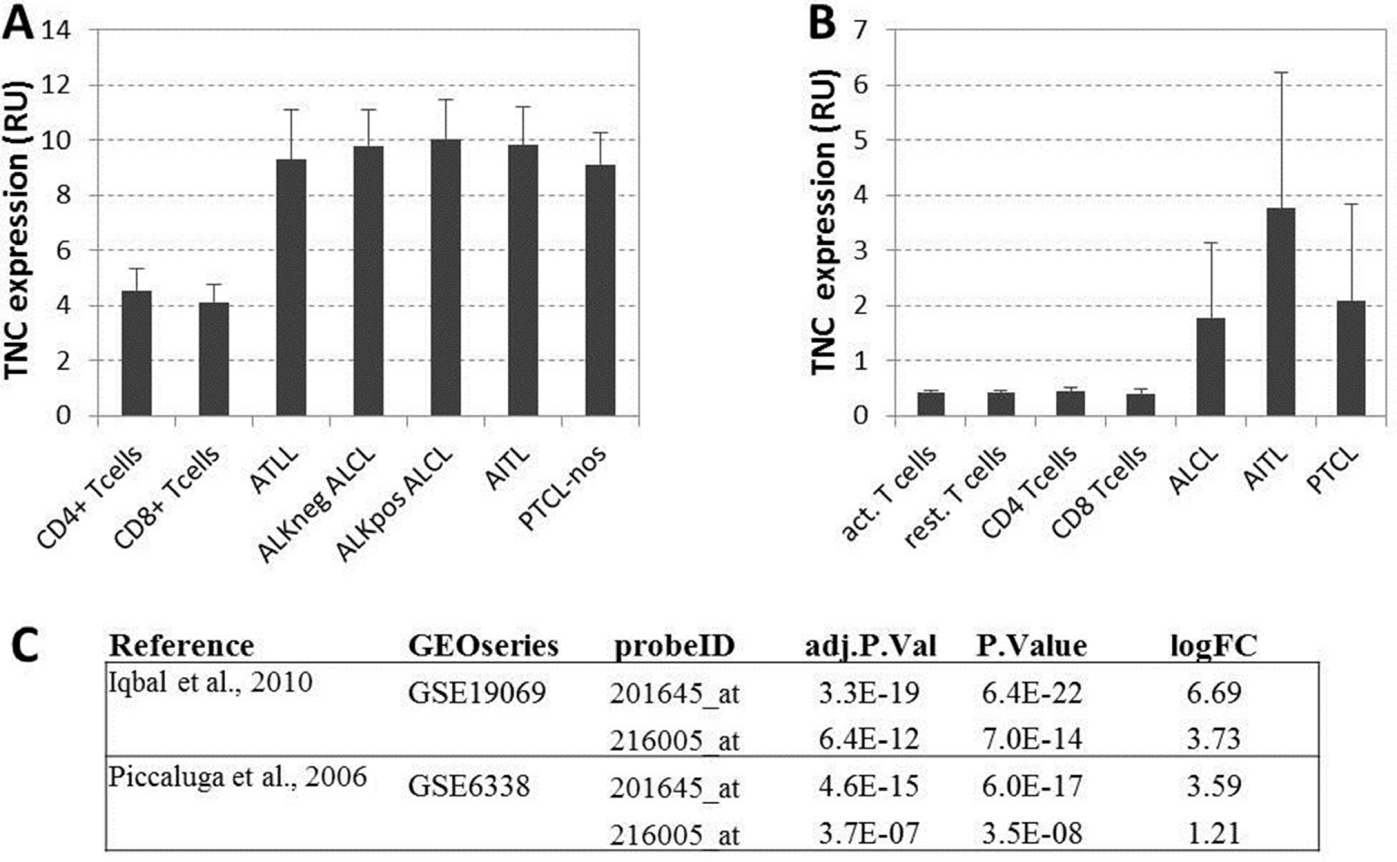
(**A**) Gene expression values from Iqbal et al. [25]: TNC average expression for resting cells and five type of lymphomas. (**B**) Gene expression values from Piccaluga et al [26]: TNC average expression for resting and activated normal CD4+ and CD8+ T cells and three types of lymphomas. All expression values are reported in relative fluorescence unit, as from original microarray datasets extracted with GEO2R functionality of the repository. (**C**) Differential expression values of TNC gene expression between normal tissues (resting or activated non lymphoma cells) and lymphomas from the two considered datasets relative to the TNC probes on the microarray: adjusted *p* values (BH correction), raw *p* Value and log fold change are shown for two TNC probes in both studies.

To further assess the expression of tenascin-C by T-cell NHL, we evaluated the presence at mRNA level of TNC using publicly available reports. Two large published dataset of PTCL were used for this analysis (GSE19069 and GSE6338) [25, 26]. Gene expression datasets were both visualized with the repository’s GEO2R feature (Supplementary Figure 2) and retrieved for local processing. Expression values of Tenascin-C microarray probes were extracted for normal and T-cell NHL samples. Overexpression of TNC was observed in all PTCL subgroups compared to normal resting and activated CD4^+^/CD8^+^T-cells in both datasets (Figure 3A–3B). Differential expression between normal T cells and aggregated PTCL groups performed with Limma-based GEO2R feature on log2 transformed data showed a statistically significant increase of TNC expression in PTCL groups with an average log fold change of 3.8 (Figure 3C).

**Figure 4:**
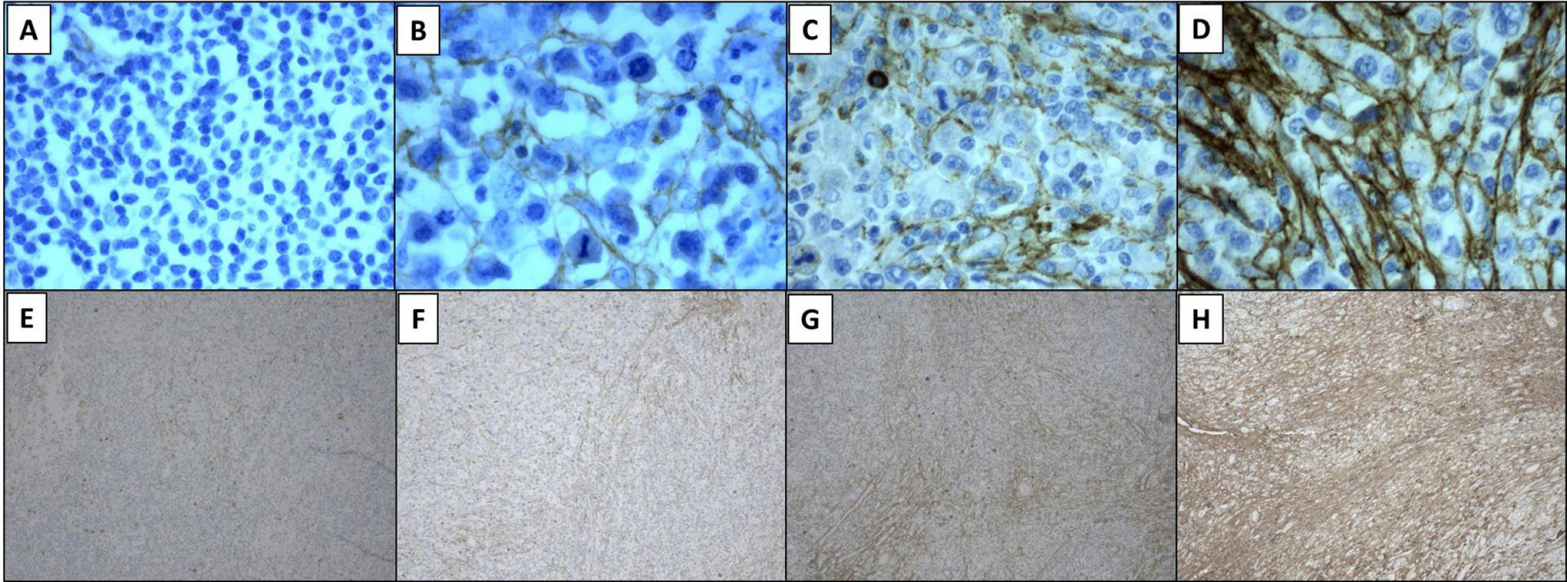
Tenascin-C expression was evaluated with tenatumomab antibody Cases were graded according to scoring intensity as no staining (panel **A**), weak (**B**), moderate (**C**) or strong (**D**). Proportion of involved areas was assessed as follows: 0 = 0% to 25% of involved areas per lesion (**E**), 1 = 26% to 50% (**F**), 2 = 51% to 75% (**G**), 3 = 76% to 100% (**H**). Magnification 40× (A–D), 10× (E–H).

## DISCUSSION

Currently, the majority of patients diagnosed with T-cell NHL are still orphan of effective treatments and rate of refractoriness to chemotherapy did not change over the last three decades [27, 28]. While Brentuximab Vedotin showed a significant activity in relapsed/refractory ALCL, with response rate up to 86% and median PFS of 13.3 months, its use in non-ALCL CD30^+^ PTCL was associated with a limited benefit [5, 29]. Among the FDA-approved drugs i.e. Romidepsin, Pralatrexate and Belinostat, response occurs in less than 30% of the cases and median PFS does not exceed 4 months [3, 4, 6]. Thus, the research of innovative therapeutic strategies is currently a major unmet need in this patient population.

In the present study, we evaluated for the first time the IHC expression of tenascin-C in T-cell NHL using Tenatumomab, a monoclonal antibody in clinical development for RIT application. To better characterize the protein expression pattern, we evaluated several aspects including staining intensity, proportion of involved areas and pattern of expression. Tenatumomab revealed the presence of tenascin-C in nearly all the cases at variable intensity. Additionally, the presence of tenascin-C, as detected by IHC, resulted in concordance with its expression at mRNA level as found by GEP analysis from independent, publicly available datasets. Results obtained by a manual scoring were validated using a digital image analysis system. Most of the systemic T-cell NHL showed both strong and diffuse tenascin-C expression, especially ALCL and AITL histology. Conversely, in MF/SS and pcALCL, staining was less intense and sparse, and tenascin-C was mainly present in the stromal architecture. Those differences in expression pattern may be related to the major tissue alteration and neoangiogenesis characterizing aggressive PTCL. Indeed, we found a particularly strong and diffuse expression of tenascin-C in AITL, which is well recognized to show the most prominent vascular component among lymphomas. We also report a possible correlation of tenascin-C immunohistochemical expression with clinical parameters in this cohort of NHL patients although this observation must be verified in larger prospective studies. We also found a differential involvement by tenascin-C among the histologies, and we described the presence of a vascular expression pattern possibly associated with survival outcomes and probably related to the different expression of tenascin-C among PTCL subgroups [30]. The patient population evaluated in this study shows a high prevalence of ALCL compared to other series [8]; this finding, probably attributable to the pattern of referral, is similar to those previously reported [9, 31].

Our findings are in line with previous reports in pathological samples of B-cell NHL and Hodgkin disease with different anti tenascin-C antibodies. The F16 anti-tenascin-C antibody (PhilogenSpA, Siena, Italy) strongly stained nearly all the cases, with tenascin-C being present in the extracellular matrix and the hyperplastic blood vessels [17]. Interestingly, in addition to NHL, tenascin-C expression has been previously reported also in the cytoplasm of neoplastic cells of several solid tumors such as breast, laryngeal or cholangiocarcinomas [32–34]. Cytoplasmic expression is often reported to be present at the invasive front of the tumor, being generally considered a marker of aggressive behavior associated with poor prognosis [33, 34]. Additionally, it has been reported secretion of tenascin-C by human carcinoma cell lines [35]. Differently from Hodgkin and B-cell NHL, we observed that tenascin-C was expressed in the cytoplasm of PTCL, in particularly in a proportion of ALCL (32%) and AITL (44%) cases, and it was associated to a diffuse stromal and perivascular tissue expression.

The clinical feasibility of targeting tenascin-C expressed in the microenvironment of B-cell NHL and Hodgkin lymphoma has been previously reported. The 81C6 antibody (Neuradiab, Bradmer Pharmaceuticals Inc., Toronto, Canada) was tested in a phase I study enrolling B-cell NHL patients [21]. Toxicity profile was limited to hematological events with 4 patients necessitating infusion of autologous bone marrow stem cells. An objective response was observed in 2 of the 9 patients, including one complete remission. More recently, the F16 antibody directed to the extra-domain A1 of tenascin-C conjugated with ^131^I (Tenarad, PhilogenSpA, Siena, Italy) was tested in 8 patients with relapsed/refractory Hodgkin lymphoma [20]. Toxicity profile was acceptable and mainly limited to hematological events, objective response was observed in two of the eight patients, including one complete remission. The anti-tenascin antibody ST2146 (Tenatumomab) has been developed to be applied in the context of the three-step Pretargeted Antibody-Guided Radio-immunotherapy (PAGRIT). This approach, exploits the tetravalency of avidin/streptavidin toward biotin and is based on the sequential administration of a biotinylated monoclonal antibody, avidin/streptavidin, and a radiolabeled biotin, leading to specific accumulation of radioactivity at the tumor site [24]. Interestingly, in a case report, biotinylated Tenatumomab monoclonal antibody and ^90^Y-biotinDOTA (^90^Y-ST2210) proved to be extremely effective in a pediatric patient with ALCL ALK positive, relapsing after allogeneic stem cell transplant [36]. However, further development of Tenatumomab in PAGRIT was hampered by regulatory hurdles. Tenatumomab has been directly conjugated with ^131^I for RIT application and is currently in phase I clinical development in tenascin-C expressing cancer (NCT02602067).

In conclusion, our data show that Tenatumomab is able to reveal the expression of tenascin-C in systemic T-cell NHL. Thus, tenascin-C represents an attractive target that sets the bases for exploring the activity of radiolabeled Tenatumomab in this orphan disease.

## MATERIALS AND METHODS

### Patients and samples

Patients with a confirmed diagnosis of T-cell NHL regularly followed at the Hematology Unit of “*Papa Giovanni XXIII*” Hospital, Bergamo were considered for this study. The diagnosis was done according to the 2008 WHO classification [7]. Clinical data were gathered from the electronic charts and our data bases. The project was approved by the Hospital Ethic Committee (authorization number 980/2014 of the 19-Jun-2014).

### Immunohistochemistry

Standard immunohistochemistry was performed on formalin fixed-paraffin embedded diagnostic samples obtained from our Department of Pathology. The length of 10% neutral-buffered formalin fixation was 6 to 48 hours, depending to dimension of the specimen and in according to ASCO/CAP Guidelines [37]. Deparaffination, target retrieval and rehydration were performed using a single run with Dako Envision Target Retrieval Solution low pH (Dako code K800521-2) in Dako Thermostated Bath (Dako PT link) at 97°C for 15 minutes. After, sections were stained by Dako Autostainer Link 48 using Dako Flex Kit (DAKO code K 800221-1). In particular, the first step was to block each section with Envision Flex Peroxidase blocking reagent for 5 minutes at room temperature and then slides were rinsed in wash buffer (Envision wash Buffer 20x; Dako code K8000). They were incubated with Tenatumomab, primary anti-human Tenascin C antibody (Sigma Tau S.p.a. R&D, Pomezia, Italy) for 30 minutes at room temperature (1:1000 dilution) and then the solution was removed by washing in buffer solution. Envision Flex HRP was added to each section and they were incubated for 20 minutes at room temperature; then the reaction was stopped by wash buffer. Afterwards Envision Flex DAB substrate was added and incubated for 10 minutes at room temperature and then slides were rinsed by wash buffer. Sections were counterstained with Envision Flex Hematoxylin (Dako code k8008) for 5 minutes and then they were washed in deionized water. Lastly, slides were dehydrated and coverslips were mounted. Negative controls included slides incubated with a non-relevant isotype-matched antibody. Slides were assessed by three independent experienced investigators (A.G., L.G.S. and L.C.) using light microscopy and staining was scored using an arbitrary scale based on four different levels “no staining”, “weak”, “moderate” and “strong” (0 to 3). A grading system was used to express the proportion of involved areas in each case, as follows: 0% to 25% of involved areas per lesion, 26% to 50%, 51% to 75% and 76% to 100%. In Figure 1 is shown a representative panel of the expression level graded in a four-point scale according to staining intensity and proportion of involved areas. Association of tenascin-C with vasculature, stroma or cytoplasm was annotated (Figure 1) [38]. All immunostained slides were digitally scanned and area of interest was calculated using Microscope Axio Imager 2 with AxioCam MRc5 and Microscope Software Axiovision (Zeiss).

### Gene expression analysis

We retrieved raw data from two publicly available gene expression dataset (Gene Expression Omnibus data sets GSE19069 and GSE6338) [25, 26]. In the two series, 183 unique cases of T-cell NHL were present, specifically 72 PTCL-not otherwise specified (NOS), 42 angioimmunoblastic lymphoma (AITL), 35 ALCLs, 20 CTCL, 12 adult T-cell leukemia/lymphoma. Expression value of tenascin-C gene (TNC) was extracted and expressed in relative fluorescence unit, as from original microarray dataset.

### Statistical analysis

All the associations between expression of tenascin-C and clinical characteristics were evaluated with Chi-squared test or Fisher’s exact test, as appropriate. Overall survival and progression-free survival were estimated by the Kaplan-Meier method and survival curves were compared applying the log-rank test. Cox proportional hazard models were used to estimate hazard ratios and 95% confidence intervals in multivariable setting. Proportional hazard assumption was verified for all estimated models. Overall survival (OS) was calculated from diagnosis to death as a result of any cause and progression-free survival (PFS) from diagnosis to disease progression or death as a result of any cause. The weighted Cohen’s kappa coefficient was calculated using Cicchetti-Allison weights to evaluate the agreement between digital and manual scoring. All reported *p*-values were two sided and the conventional 5% significance level was fixed. Statistical analysis was performed using SAS software (version 9.4).

## SUPPLEMENTARY MATERIALS FIGURES AND TABLES



## Abbreviations

AITL: Angioimmunoblastic lymphoma
ALCL: Anaplastic large cell lymphoma
ALK: Anaplastic large cell lymphoma kinase
CTCL: Cutaneous T-cell lymphoma
EGF: Epidermal growth factor
IHC: Immunohistochemistry
IPI: International prognostic score
NHL: Non-Hodgkin lymphoma
OS: Overall survival
PAGRIT: Pretargeted Antibody-Guided Radioimmuno-Therapy
PFS: Progression-free survival
PTCL: Peripheral T-cell lymphoma
RIT: Radio-immunotherapy
TNC: Tenascin-C gene

## References

[1] Chen BJ, Chapuy B, Ouyang J, Sun HH, Roemer MG, Xu ML, Yu H, Fletcher CD, Freeman GJ, Shipp MA, Rodig SJ (2013). PD-L1 expression is characteristic of a subset of aggressive B-cell lymphomas and virus-associated malignancies. Clin Cancer Res.

[2] Batlevi CL, Matsuki E, Brentjens RJ, Younes A (2016). Novel immunotherapies in lymphoid malignancies. Nat Rev Clin Oncol.

[3] O'Connor OA, Pro B, Pinter-Brown L, Bartlett N, Popplewell L, Coiffier B, Lechowicz MJ, Savage KJ, Shustov AR, Gisselbrecht C, Jacobsen E, Zinzani PL, Furman R (2011). Pralatrexate in patients with relapsed or refractory peripheral T-cell lymphoma: results from the pivotal PROPEL study. J Clin Oncol.

[4] Coiffier B, Pro B, Prince HM, Foss F, Sokol L, Greenwood M, Caballero D, Borchmann P, Morschhauser F, Wilhelm M, Pinter-Brown L, Padmanabhan S, Shustov A (2012). Results from a pivotal, open-label, phase II study of Romidepsin in relapsed or refractory peripheral T-cell lymphoma after prior systemic therapy. J Clin Oncol.

[5] Pro B, Advani R, Brice P, Bartlett NL, Rosenblatt JD, Illidge T, Matous J, Ramchandren R, Fanale M, Connors JM, Yang Y, Sievers EL, Kennedy DA (2012). Brentuximab vedotin (SGN-35) in patients with relapsed or refractory systemic anaplastic large-cell lymphoma: results of a phase II study. J Clin Oncol.

[6] O'Connor OA, Horwitz S, Masszi T, Van Hoof A, Brown P, Doorduijn J, Hess G, Jurczak W, Knoblauch P, Chawla S, Bhat G, Choi MR, Walewski J (2015). Belinostat in patients with relapsed or refractory peripheral T-cell lymphoma: results of the pivotal Phase II BELIEF (CLN-19) study. J Clin Oncol.

[7] Swerdlow SH, Campo E, Harris NL, Jaffe ES, Pileri SA, Stein H, Thiele J, Vardiman JW (2008). Pathology and genetics of tumours of hematopoietic and lymphoid tissues. World Health Organization classification of tumours.

[8] Vose J, Armitage J, Weisenburger D (2008). International peripheral T-cell and natural killer/T-cell lymphoma study: pathology findings and clinical outcomes. J Clin Oncol.

[9] Gritti G, Boschini C, Rossi A, Delaini F, Grassi A, Algarotti A, Mico C, Trezzi R, Gianatti A, Barbui AM, Rambaldi A (2015). Primary treatment response rather than front line stem cell transplantation is crucial for long term outcome of peripheral T-cell lymphomas. PLoS One.

[10] Brellier F, Chiquet-Ehrismann R (2012). How do tenascins influence the birth and life of a malignant cell?. J Cell Mol Med.

[11] Jahkola T, Toivonen T, Nordling S, von Smitten K, Virtanen I (1998). Expression of tenascin-C in intraductal carcinoma of human breast: relationship to invasion. Eur J Cancer.

[12] Ghert MA, Qi WN, Erickson HP, Block JA, Scully SP (2001). Tenascin-C splice variant adhesive/anti-adhesive effects on chondrosarcoma cell attachment to fibronectin. Cell Struct Funct.

[13] Zagzag D, Capo V (2002). Angiogenesis in the central nervous system: a role for vascular endothelial growth factor/vascular permeability factor and tenascin-C. Common molecular effectors in cerebral neoplastic and non-neoplastic “angiogenic diseases”. Histol Histopathol.

[14] Puente Navazo MD, Valmori D, Ruegg C (2001). The alternatively spliced domain TnFnIII A1A2 of the extracellular matrix protein tenascin-C suppresses activation-induced T lymphocyte proliferation and cytokine production. J Immunol.

[15] Jaspars LH, Bloemena E, Bonnet P, Van der Valk P, Meijer CJ (1995). Distribution of extracellular matrix components and their receptors in human lymphoid tissue and B-cell non-Hodgkin lymphomas. Histopathology.

[16] Vacca A, Ribatti D, Fanelli M, Costantino F, Nico B, Di Stefano R, Serio G, Dammacco F (1996). Expression of tenascin is related to histologic malignancy and angiogenesis in b-cell non-Hodgkin's lymphomas. Leuk Lymphoma.

[17] Schliemann C, Wiedmer A, Pedretti M, Szczepanowski M, Klapper W, Neri D (2009). Three clinical-stage tumor targeting antibodies reveal differential expression of oncofetal fibronectin and tenascin-C isoforms in human lymphoma. Leuk Res.

[18] Rizzieri DA, Wadleigh M, Wikstrand CJ, Mann KP, Sen F, Peterson BL, Niedzwiecki D, Proia AD, Bigner DD (2005). Tenascin and microvessel stromal changes in patients with non-Hodgkin's lymphoma are isolated to the sites of disease and vary in correlation to disease activity. Leuk Lymphoma.

[19] Paganelli G, Grana C, Chinol M, Cremonesi M, De Cicco C, De Braud F, Robertson C, Zurrida S, Casadio C, Zoboli S, Siccardi AG, Veronesi U (1999). Antibody-guided three-step therapy for high grade glioma with yttrium-90 biotin. Eur J Nucl Med.

[20] Aloj L, D'Ambrosio L, Aurilio M, Morisco A, Frigeri F, Caraco C, Di Gennaro F, Capobianco G, Giovannoni L, Menssen HD, Neri D, Pinto A, Lastoria S (2014). Radioimmunotherapy with Tenarad, a 131I-labelled antibody fragment targeting the extra-domain A1 of tenascin-C, in patients with refractory Hodgkin's lymphoma. Eur J Nucl Med Mol Imaging.

[21] Rizzieri DA, Akabani G, Zalutsky MR, Coleman RE, Metzler SD, Bowsher JE, Toaso B, Anderson E, Lagoo A, Clayton S, Pegram CN, Moore JO, Gockerman JP (2004). Phase 1 trial study of 131I-labeled chimeric 81C6 monoclonal antibody for the treatment of patients with non-Hodgkin lymphoma. Blood.

[22] De Santis R, Anastasi AM, D'Alessio V, Pelliccia A, Albertoni C, Rosi A, Leoni B, Lindstedt R, Petronzelli F, Dani M, Verdoliva A, Ippolito A, Campanile N (2003). Novel antitenascin antibody with increased tumour localisation for Pretargeted Antibody-Guided RadioImmunoTherapy (PAGRIT). Br J Cancer.

[23] De Santis R, Albertoni C, Petronzelli F, Campo S, D'Alessio V, Rosi A, Anastasi AM, Lindstedt R, Caroni N, Arseni B, Chiodi P, Verdoliva A, Cassani G (2006). Low and high tenascin-expressing tumors are efficiently targeted by ST2146 monoclonal antibody. Clin Cancer Res.

[24] Petronzelli F, Pelliccia A, Anastasi AM, D'Alessio V, Albertoni C, Rosi A, Leoni B, De Angelis C, Paganelli G, Palombo G, Dani M, Carminati P, De Santis R (2005). Improved tumor targeting by combined use of two antitenascin antibodies. Clin Cancer Res.

[25] Iqbal J, Weisenburger DD, Greiner TC, Vose JM, McKeithan T, Kucuk C, Geng H, Deffenbacher K, Smith L, Dybkaer K, Nakamura S, Seto M, Delabie J (2010). Molecular signatures to improve diagnosis in peripheral T-cell lymphoma and prognostication in angioimmunoblastic T-cell lymphoma. Blood.

[26] Piccaluga PP, Agostinelli C, Califano A, Rossi M, Basso K, Zupo S, Went P, Klein U, Zinzani PL, Baccarani M, Dalla Favera R, Pileri SA (2007). Gene expression analysis of peripheral T cell lymphoma, unspecified, reveals distinct profiles and new potential therapeutic targets. J Clin Invest.

[27] O'Connor OA, Bhagat G, Ganapathi K, Pedersen MB, D'Amore F, Radeski D, Bates SE (2014). Changing the paradigms of treatment in peripheral T-cell lymphoma: from biology to clinical practice. Clin Cancer Res.

[28] Tarella C, Gueli A, Delaini F, Rossi A, Barbui AM, Gritti G, Boschini C, Caracciolo D, Bruna R, Ruella M, Gottardi D, Passera R, Rambaldi A (2014). Rate of primary refractory disease in B and T-cell non-Hodgkin's lymphoma: correlation with long-term survival. PLoS One.

[29] Horwitz SM, Advani RH, Bartlett NL, Jacobsen ED, Sharman JP, O'Connor OA, Siddiqi T, Kennedy DA, Oki Y (2014). Objective responses in relapsed T-cell lymphomas with single-agent brentuximab vedotin. Blood.

[30] Cardesa-Salzmann TM, Colomo L, Gutierrez G, Chan WC, Weisenburger D, Climent F, Gonzalez-Barca E, Mercadal S, Arenillas L, Serrano S, Tubbs R, Delabie J, Gascoyne RD (2011). High microvessel density determines a poor outcome in patients with diffuse large B-cell lymphoma treated with rituximab plus chemotherapy. Haematologica.

[31] Schmitz N, Trumper L, Ziepert M, Nickelsen M, Ho AD, Metzner B, Peter N, Loeffler M, Rosenwald A, Pfreundschuh M (2010). Treatment and prognosis of mature T-cell and NK-cell lymphoma: an analysis of patients with T-cell lymphoma treated in studies of the German High-Grade Non-Hodgkin Lymphoma Study Group. Blood.

[32] Ishihara A, Yoshida T, Tamaki H, Sakakura T (1995). Tenascin expression in cancer cells and stroma of human breast cancer and its prognostic significance. Clin Cancer Res.

[33] Aishima S, Taguchi K, Terashi T, Matsuura S, Shimada M, Tsuneyoshi M (2003). Tenascin expression at the invasive front is associated with poor prognosis in intrahepatic cholangiocarcinoma. Mod Pathol.

[34] Yoshida T, Yoshimura E, Numata H, Sakakura Y, Sakakura T (1999). Involvement of tenascin-C in proliferation and migration of laryngeal carcinoma cells. Virchows Arch.

[35] Kawakatsu H, Shiurba R, Obara M, Hiraiwa H, Kusakabe M, Sakakura T (1992). Human carcinoma cells synthesize and secrete tenascin *in vitro*. Jpn J Cancer Res.

[36] Palumbo G, Grana CM, Cocca F, De Santis R, Del Principe D, Baio SM, Mei R, Paganelli G (2007). Pretargeted antibody-guided radioimmunotherapy in a child affected by resistant anaplastic large cell lymphoma. Eur J Haematol.

[37] Hammond ME, Hayes DF, Dowsett M, Allred DC, Hagerty KL, Badve S, Fitzgibbons PL, Francis G, Goldstein NS, Hayes M, Hicks DG, Lester S, Love R (2010). American Society of Clinical Oncology/College of American Pathologists guideline recommendations for immunohistochemical testing of estrogen and progesterone receptors in breast cancer. Arch Pathol Lab Med.

[38] Spenle C, Gasser I, Saupe F, Janssen KP, Arnold C, Klein A, van der Heyden M, Mutterer J, Neuville-Mechine A, Chenard MP, Guenot D, Esposito I, Slotta-Huspenina J (2015). Spatial organization of the tenascin-C microenvironment in experimental and human cancer. Cell Adh Migr.

