# Correction: Evaluation of tenascin-C by tenatumomab in T-cell non-Hodgkin lymphomas identifies a new target for radioimmunotherapy

**DOI:** 10.18632/oncotarget.25064

**Published:** 2018-03-30

**Authors:** Giuseppe Gritt, Andrea Gianatt, Fiorella Petronzeii, Rita De Santis, Chiara Pavoni, Riccardo Lorenzo Rossi, Laura Cattaneo, Luigi Giusto Spagnoli, Silvia Ferrar, Andrea Ross, Anna Maria Barbu, Alessandro Rambald

**Affiliations:** ^1^ Hematology and Bone Marrow Transplant Units, Ospedale Papa Giovanni XXIII, Bergamo, Italy; ^2^ Pathology Unit, Ospedale Papa Giovanni XXII, Bergamo, Italy; ^3^ Sigma Tau S.p.A. Biotech Products Rand D, Pomezia, Italy; ^4^ Bioinformatics, Istituto Nazionale Genetica Molecolare, Milan, Italy; ^5^ Department of Biomedicine and Prevention, Universita di Roma Tor Vergata, Rome, Italy; ^6^ Department of Oncology and Oncohematology, Universita degli Studi di Milano, Milan, Italy

**This article has been corrected:** The online version of figure 3 has been corrected:

**Figure 3:**
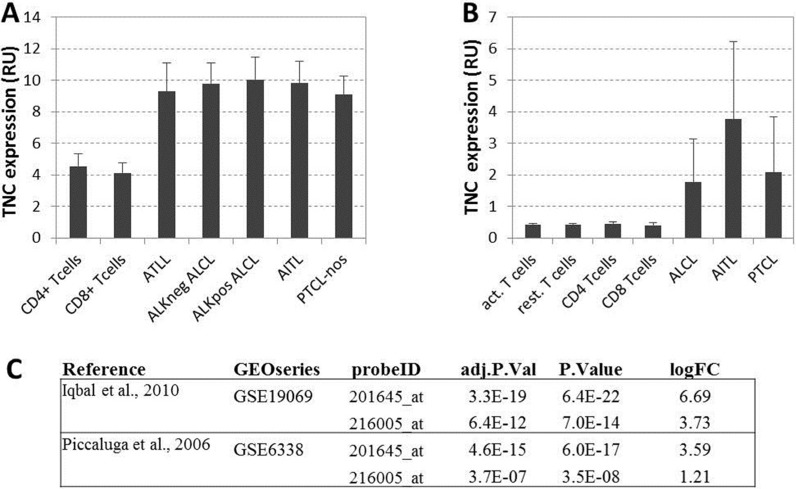
(A) Gene expression values from Iqbal et al. [25]: TNC average expression for resting cells and five type oflymphomas. **(B)** Gene expression values from Piccaluga et al [26]: TNC average expression for resting and activated normal CD4+ and CDS+ T cells and three types oflymphomas. All expression values are reported in relative fluorescence unit, as from original microarray datasets extracted with GE02R fimctionality of the repository. (C) Differential expression values ofTNC gene expression between normal tissues (resting or activated non lymphoma cells) and lymphomas from the two considered datasets relative to the TNC probes on the microarray: adjusted *p* values (BH correction), raw *p* Value and log fold change are shown for two TNC probes in both studies.

Original article: Oncotarget. 2018; 9:9766-9775. https://doi.org/10.18632/oncotarget.23919

