# Supplementary material for: Evaluation of tenascin-C by tenatumomab in T-cell non-Hodgkin lymphomas identifies a new target for radioimmunotherapy
Source: Oncotarget. 2018 Jan 3;9(11):9766–75. doi: 10.18632/oncotarget.23919 (PMC5839400; doi:10.18632/oncotarget.23919)
Supplement: Supplementary file 1 [file oncotarget-09-9766-s001.pdf]

Evaluation of tenascin-C by tenatumomab in T-cell non-Hodgkin lymphomas identifies a new target for radioimmunotherapy

SUPPLEMENTARY MATERIALS

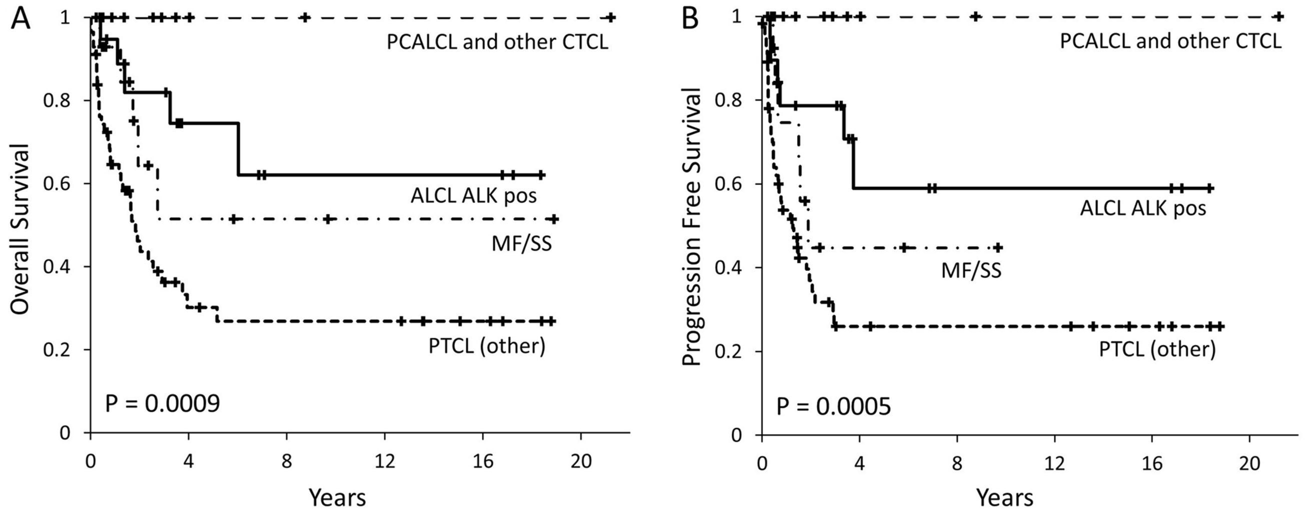

Supplementary Figure 1: Overall survival (**A**) and progression free survival (**B**) according to diagnosis in the whole cohort. ALCL ALK+:anaplastic large cell lymphoma ALK positive (N = 19); PTCL others: peripheral T-cell lymphoma with diagnosis different than ALCL ALK positive (N = 56) ; MF/SS: Mycosis fungoides/Sézary syndrome (N = 14); PCALCL: primary cutaneous ALCL (N = 6); Others: includesCD4+ small/medium cell primary cutaneous lymphoma (N = 5).

A Iqbal et al., GSE19069

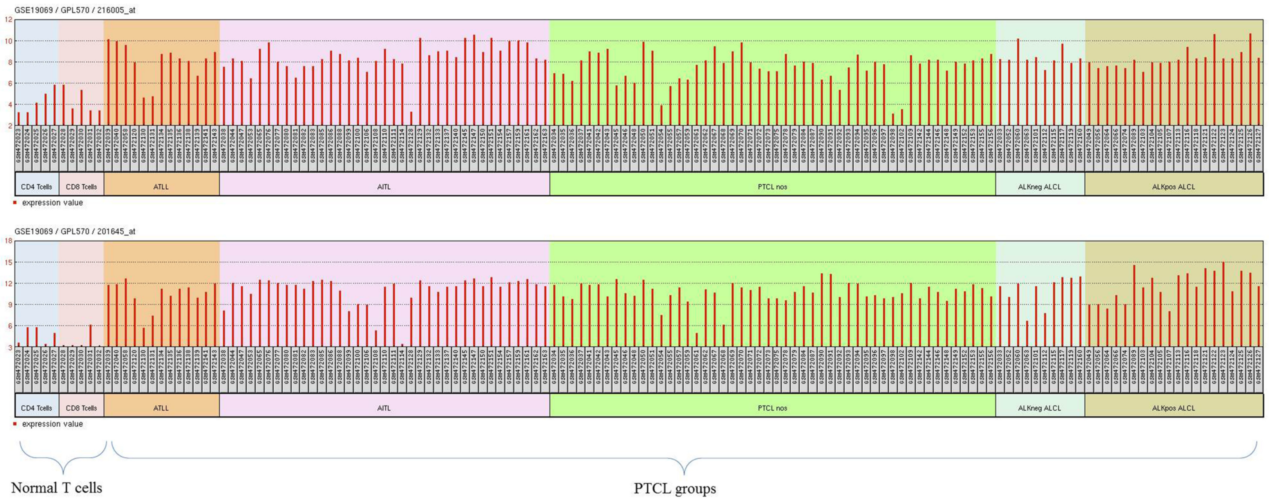

B Piccaluga et al., GSE6338

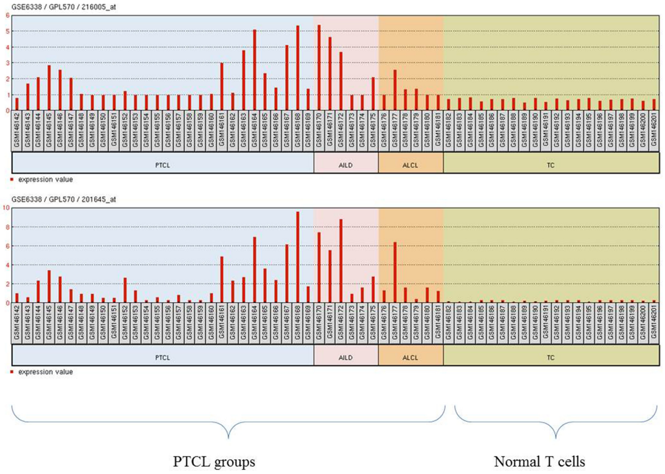

Supplementary Figure 2: Detailed view of expression values for TNC probes (210065_at and 201645_at) in all considered normal and lymphoma samples for (**A**) Iqbal et al. 2010 [25] GSE19069 dataset and, (**B**) Piccaluga et al. 2007 [26] dataset GSE6338.

Supplementary Table 1: Proportion of involved areas according to method of detection

Histology	N	Digital Determination - Continuous (%)	Digital Determination - Score (%)				Manual Determination - Score (%)			
		Median (range)	0- 25	26–50	51–75	76–100	0- 25	26–50	51–75	76–100
**Precursor (lymphoblastic) T-cell NHL**	**3**	**67 (0–75)**	**1**	**0**	**2**	**0**	**1**	**0**	**2**	**0**
Peripheral T-cell lymphoma, not otherwise specified	20	44 (0–83)	5	9	5	1	5	9	5	1
Anaplastic large cell lymphoma, ALK negative	21	67 (0–90)	2	2	14	3	2	2	14	3
Anaplastic large cell lymphoma, ALK positive	19	53 (22–85)	0	8	9	2	0	8	9	2
Angioimmunoblastic T-cell lymphoma	9	60 (30–90)	0	2	6	1	0	2	6	1
Enteropathy-associated T-cell lymphoma	2	42 (0–84)	1	0	0	1	1	0	0	1
Hepatosplenic T-cell lymphoma	1	38 (38–38)	0	1	0	0	0	1	0	0
**PTCL (subtotal)**	**72**	**58 (0–90)**	**8**	**22**	**34**	**8**	**8**	**22**	**34**	**8**
Mycosis fungoides	13	34 (0–70)	1	9	3	0	1	9	3	0
*Primary cutaneous anaplastic large cell lymphom*a	6	56 (0–73)	1	2	3	0	1	2	3	0
CD4^{+} small/medium cell primary cutaneous lymphoma	5	0 (0–45)	3	2	0	0	3	2	0	0
Sézary syndrome	1	56 (56–56)	0	1	0	0	0	1	0	0
**CTCL (subtotal)**	**25**	**34 (0–73)**	**5**	**14**	**6**	**0**	**5**	**14**	**6**	**0**
**Total**	**100**	**51 (0–90)**	**14**	**36**	**42**	**8**	**14**	**36**	**42**	**8**

Legend: NHL: non-Hodgkin lymphoma; PTCL: peripheral T-cell lymphoma; CTCL: cutaneous T-cell lymphoma.

Supplementary Table 2: Multivariate analysis for PFS and OS in systemic T-cell NHL patients with digital image analysis ($N = 75$)

Factors	Multivariate for PFS				Multivariate for OS			
	HR (95% Cl)	*P* value	HR (95% Cl)	*P* value	HR (95% Cl)	*P* value	HR (95% Cl)	*P* value
**Proportion of involved areas** (%) (by Digital Image Analysis)								
0–50	1.00				1.00			
51–100	**0.49 (0.25–0.95)**	**0.0337**			0.56 (0.28–1.12)	0.1034		
**Proportion of involved areas (continuous value)** (by Digital Image Analysis)			0.32 (0.09–1.17)	0.0870			0.31 (0.08–1.23)	0.0944
**Age**								
≤ 60	1.00		1.00		1.00		1.00	
> 60	1.54 (0.70–3.38)	0.2866	1.36 (0.62–2.99)	0.4422	1.81 (0.81–4.05)	0.1485	1.65 (0.75–3.61)	0.2144
**Gender**								
Female	1.00		1.00		1.00		1.00	
Male	1.13 (0.57–2.24)	0.7366	1.19 (0.60–2.34)	0.6184	1.48 (0.73–3.02)	0.2797	1.57 (0.76–3.21)	0.2203
**IPI**								
Low	1.00		1.00		1.00		1.00	
Interm-Low	1.67 (0.59–4.70)	0.3330	1.75 (0.62–4.97)	0.2927	1.98 (0.67–5.84)	0.2155	2.01 (0.68–5.95)	0.2069
Interm-High	1.78 (0.69–4.77)	0.2481	1.80 (0.65–4.94)	0.2555	1.87 (0.64–5.51)	0.2542	1.80 (0.60–5.39)	0.2964
High	**4.11 (1.61–10.52)**	**0.0032**	**4.09 (1.53–10.92)**	**0.0049**	**5.12 (1.93–13.54)**	**0.0010**	**5.14 (1.91–13.89)**	**0.0012**

Legend: NHL: non-Hodgkin lymphoma; PFS: progression free survival; OS: overall survival.
